# Predictions through evidence accumulation over time

**DOI:** 10.1038/s41598-017-18802-z

**Published:** 2018-01-11

**Authors:** Álvaro Darriba, Florian Waszak

**Affiliations:** 1 Université Paris Descartes, Sorbonne Paris Cité, 75006 Paris, France; 2 Centre National de la Recherche Scientifique, Laboratoire Psychologie de la Perception, Unité Mixte de Recherche 8242, 75006 Paris, France

## Abstract

It has been proposed that the brain specializes in predicting future states of the environment. These predictions are probabilistic, and must be continuously updated on the basis of their mismatch with actual evidence. Although electrophysiological data disclose neural activity patterns in relation to predictive processes, little is known about how this activity supports prediction build-up through evidence accumulation. Here we addressed this gap. Participants were required to make moment-by-moment predictions about stimuli presented in sequences in which gathering evidence from previous items as they were presented was either possible or not. Two event-related potentials (ERP), a frontocentral P2 and a central P3, were sensitive to information accumulation throughout the sequence. Time-frequency (TF) analyses revealed that prediction build-up process also modulated centrally distributed theta activity, and that alpha power was suppressed in anticipation to fully predictable stimuli. Results are in agreement with the notion of predictions as probability distributions and highlight the ability of observers to extract those probabilities in a changing environment and to adjust their predictions consequently.

## Introduction

Contextual information allows us to generate predictions about upcoming events. According to predictive coding models^1,2^, predictions are represented as probability distributions that are continuously compared with actual evidence and adjusted accordingly. Predictions are primarily based on our general knowledge and experience, i.e., on global probabilities about how events succeed in a given context. Importantly, this global knowledge is modulated by local events that influence the probabilities of other events and compel predictions to be fine-tuned on a moment-by-moment basis. Thereby, in a stable context predictions are expected to become progressively more accurate as more information about the regularities of the environment is provided over time. Surprisingly, this issue has been rarely addressed in previous studies^3,4^. The present work is meant to fill this gap. Its main goal was to identify and dissociate differential scalp-recorded, processing-related voltage changes (event-related potentials, ERP) and oscillations in the electroencephalogram (EEG) and to examine their functional significance in the build-up of predictions as evidence accumulates over time.

Most previous research has focused on brain activity related to either the fulfilment or the violation of predictions made on the basis of strong contextual regularities, usually the continuous repetition of a stimulus^5,6^, the presentation of predictable pairs of stimuli where the first predicts the second^7^ or a fixed and predetermined sequence of stimuli^8,9^. All these experiments have in common that expectations involve a binary prediction, i.e., an event is expected to occur or not, and subjects know, in case it occurs, what it will exactly consist of, so they can hypothetically generate a strong and precise template and compare the upcoming event with it. In our paradigm, by contrast, participants were presented with either predictable or unpredictable sequences and were asked to figure out how these sequences will evolve over time. In the unpredictable condition every stimulus was presented pseudo-randomly and no successful predictions about the upcoming stimuli were possible, although participants were not aware of that. In the predictable conditions, however, stimuli succeeded according to a rotation rule, so that participants were expected to steadily sharpen their predictions as more evidence was provided and to perfectly anticipate the last stimulus in the series. Thus, unlike previous studies in which predictions are generated on the basis of a global knowledge about the probability of events to occur, or locally based on the immediately preceding stimulus, here accurate predictions were progressively constructed by accumulating information while observing the development of the stimuli stream over time.

A number of ERP components and oscillations have been linked to prediction in non-accumulative experimental contexts, but it is unclear how they relate to evidence accumulation for prediction build-up. Nevertheless, it is possible to formulate hypotheses about potential electrophysiological markers of evidence accumulation for prediction formation on the basis of results obtained in close research contexts, such as perceptual decision-making. Different EEG studies have identified ERP and oscillations likely reflecting such an information gathering process for decision-making. This is the case of P2 and P3 ERP components and theta-band oscillations. Regarding P2, recent literature suggests that a frontally distributed component of that family peaking around 350 ms post-stimulus, the prefrontal P2 or pP2, is associated with evidence accumulation for decision-making^10–12^. More specifically, this component has been suggested to represent the final stage of the decision-making process, once enough action-related information is accumulated, before the emission of a response^12,13^, and to be itself associated with the evidence accumulation process^10^. Moreover, P3, a positive deflection in the ERP waveform peaking between 300 and 800 ms and with a broad but varying topographical distribution depending on the task employed, is one of the most extensively studied ERP components and has been observed in multiple experimental contexts^14^. Although it has been related to a wide variety of processes, including context updating^15^, surprise processing^16^, or decision confidence^17^, its functional significance remains indefinite. In the context of decision-making, a number of experiments have described steady amplitude increases of the centroparietal P3 ERP component as a function of evidence accumulation^18,19^. These increases are thought to reflect the operation of a mechanism dedicated to gathering and integrating accrued sensory evidence for decision formation^20^. Finally, theta oscillations have been implicated in various aspects of decision-making, such as decision confidence^21^ and error monitoring^22^. Interestingly for the goals of the present research, an increase of neural activity in the theta frequency range has been found to accompany the process of decision formation, which suggests that theta oscillations are crucial in the accumulation of evidence in decision-making tasks^23–26^. Actually, neural activity in the theta frequency range has been suggested to coordinate and integrate information from various sources in a variety of tasks^27,28^. Altogether, evidence suggests a role of theta oscillations in the implementation of a general, multipurpose information accumulation mechanism in the brain.

As in perceptual decision-making, prediction build-up requires evidence accumulation over time. Actually, decisions are strongly shaped by the predictability of the upcoming event^29,30^: the accumulation of sensory evidence over time increases its predictability and consequently the accuracy of the decisions made about it^31^. However, while in decision-making sensory evidence is accumulated and compared against the current sensory input in order to select a specific action^23^, prediction build-up involves gathering evidence for anticipating an event that has not been presented yet. Although this information accumulation can be also used for preparing a response in anticipation for an event to occur, and in fact response speed and accuracy have been consistently employed for evaluating prediction, in the present experiment we were interested in studying the information accumulation process itself in the absence of an overt response. A similar approach was successfully employed in our lab in the past^8,32^ for the study of prediction-related neural activity, and constitutes an important aspect of our design since both the P2 and P3 components, that are of key interest here, have been shown to be strongly linked to response-related processing^10,12,33^ and might therefore be differentially modulated in the different experimental conditions if a response was required, making it more difficult to disentangle evidence accumulation from response preparation processes.

Previous research has suggested the existence of an evidence-accumulating decision process, evident even in the absence of overt action, domain general and independent of sensory modality and stimulus features^19^. We hypothesize that such an evidence accumulator supporting decision processing may also subserve prediction build-up. As stated before, in the present experiment participants were required to make moment-by-moment predictions about stimuli presented in sequences in which accumulating evidence from previous items as they were presented was either possible or not. With the aim of objectively defining evidence accumulation, we followed previous studies^16,29,30^ for, within the framework of Shannon’s information theory^34^, computing at each time point in the sequences surprise, a measure of the improbability of an event, and the predictive information on the forthcoming stimulus, which measures how the accumulation of evidence from preceding stimuli reduces this surprise^30^. Based on the results described above, we focused on P2 and P3 ERP components and on theta oscillations as potential electrophysiological correlates of the evidence accumulation process for prediction generation. If these ERP and oscillations actually reflect a general-purpose evidence-accumulation mechanism operating in this context, we expected them to be modulated coherently with predictive information accumulation throughout the predictable sequences, and to show no such modulations in the unpredictable series, in which building-up an evidence-based prediction was not possible. In addition, since in predictive contexts P3 has been shown to reflect predictions based on probabilities^16^, and theta oscillations have been suggested to mark the utilization of prediction error (PE) in the adjustment of predictions^32,35–37^, we expected P3 and theta oscillations to accordingly show discrete enhancements in response to low probability, surprising events.

## Results

In our experiment participants were presented with series of four stimuli (S1-S2-S3-S4) displayed sequentially. Stimuli consisted of a clock-like circle with a thick line inside pointing to one of eight possible directions. Each sequence could be either predictable or unpredictable. When unpredictable, the clock’s hand in S1, S2, S3 and S4 pointed to random positions (Unpredictable condition). When predictable, the hand steadily rotated either clockwise or anticlockwise over the sequence (Predictable-A condition) or, with equal probability, changed rotation direction in S3 (Predictable-B condition). We used information theory for parameterizing the information structure of the task with the aim of objectively defining *PE* and *accumulation of predictive information*. PE can be estimated by calculating surprise, a measure of the uncertainty of an event^38^. Predictive information is a measure of uncertainty reduction due to the knowledge of the preceding events. It is related to the conditional probability of each stimulus to be presented as a function of the stimuli that were presented before in the series, and quantifies the amount of information available at a given time for predicting which the forthcoming stimulus will be^30^. A priori calculations of these measures allowed us to design the sequences in such a way that predictability of the stimuli progressively increased over the sequences in the Predictable-A and Predictable-B conditions, so that only the last stimulus (S4) was predictable on the basis of the knowledge about the stimuli preceding it. Accumulation of predictive information was not possible in the Unpredictable condition. For a detailed description of the task, see the Methods section.

### Event-related potentials

**Figure 1:**
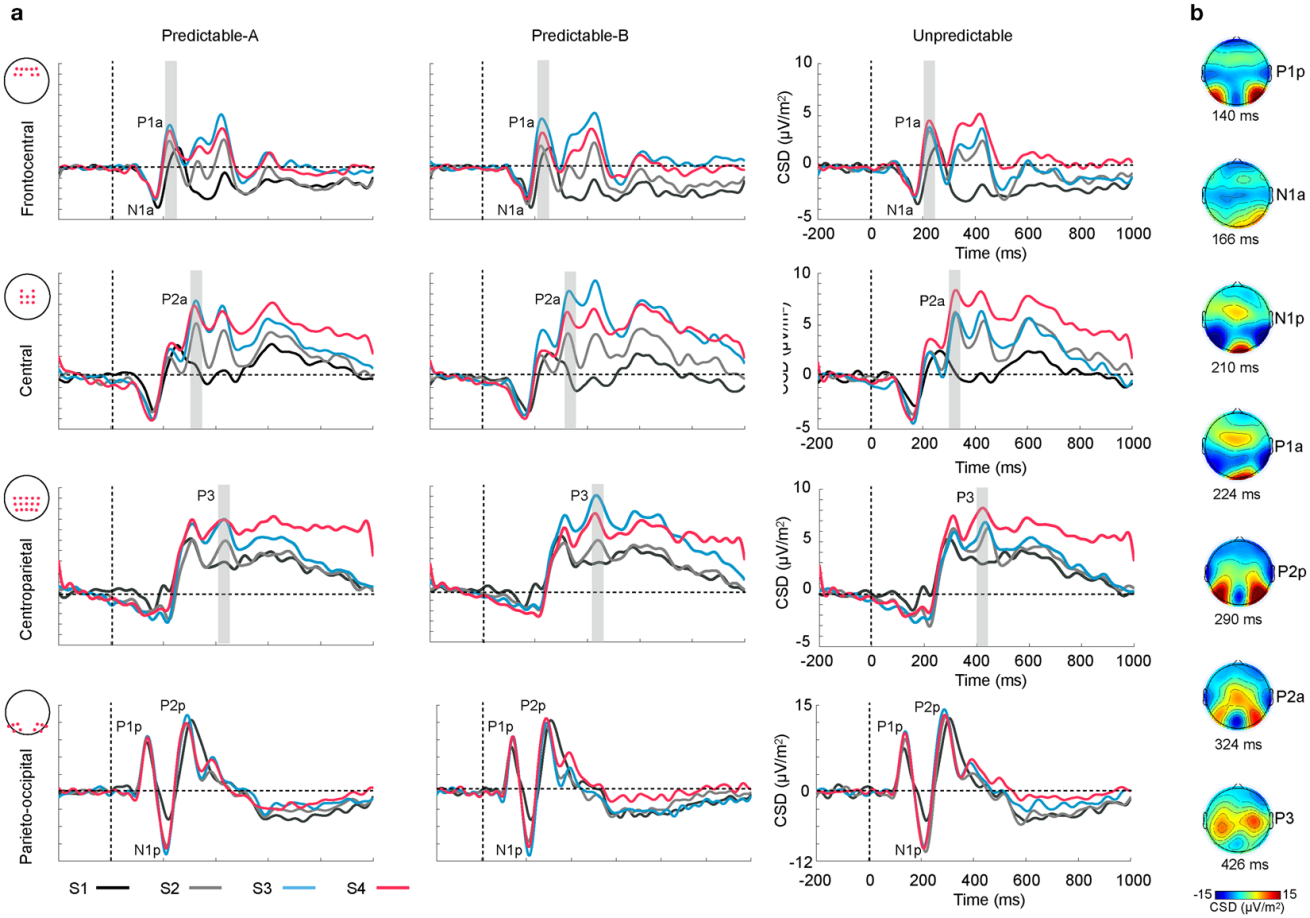
(**a**) Grand-average ERP waveforms recorded at frontocentral, central, centroparietal and lateral parieto-occipital ROIs for each stimulus in the sequence in the Predictable-A, Predictable-B and Unpredictable conditions. Electrodes included in the ROI are displayed next to the corresponding label. Waveforms were low-pass filtered at 30 Hz for presentation purposes. Grey bars highlight P1a, P2a and P3 ERPs in the Predictable-A and Predictable-B conditions, where analyses revealed significant differences throughout the sequence. (**b**) Condition-averaged topography plots depicting the voltage distribution over the scalp of each of the components labelled in Fig. 1a at the time they peaked.

Figure 1 depicts the ERP waveforms at frontocentral, central, centroparietal and parieto-occipital locations for each of the three conditions and the main components observed (a), together with the topographic maps corresponding to each of those components (b). The following components were detected according to their peak latency and topography: an early posterior positivity (P1p, peaking at 140 ms), maximal at parieto-occipital electrodes; an anterior negativity (N1a, 166 ms), maximal at central locations; a posterior negativity (N1p, 210 ms) maximal at parieto-occipital sites; an anterior positivity (P1a, 224 ms) peaking at frontocentral electrodes; a posterior positivity (P2p, 290 ms) with a strong bilateral parieto-occipital distribution; an anterior positivity (P2a, 324 ms), maximal at central sites and a central, bilateral positivity (P3, 426 ms) maximal at central and centroparietal sites.

Description of ERP results are restricted to the P2a and P3 components, which have been linked in the literature to evidence accumulation and predictive processes, and to the anterior P1a, since it exhibited evident differences between conditions in the waveforms. No significant effects were found in the analyses of the other components. Analysis were performed on clusters of relevant electrodes (regions of interest, ROI) identified for each component on the basis of both the grand average visual detection of the electrodes in which it showed maximal amplitude and its topographical distribution on the scalp. Following this procedure, P1a, P2a and P3 were measured and analysed on frontocentral, central and centroparietal ROIs respectively (see Fig. 1a,b, and the Methods section). Analyses were performed on each component separately via repeated-measures ANOVA with Condition (Predictable-A, Predictable-B, Unpredictable) and Stimulus (S1, S2, S3, S4) as factors.

P1a peaked at 224 ms post-stimulus and exhibited a predominantly frontal scalp distribution. In Fig. 1a and b, it is highlighted at frontal electrodes. A significant main effect for STIMULUS (F(3,42) = 8.89, p = 0.004, partial η^2^ = 0.39) and a significant two-way CONDITION × STIMULUS interaction (F(6,84) = 2.46, p = 0.031, partial η^2^ = 0.15) were found. The assumption of sphericity was not met for the main effect of STIMULUS (Χ^2^(5) = 23.74, p = 0.0001). Post-hoc analyses indicated that in the unpredictable sequences P1a amplitude was significantly smaller in response to S1 than to the other three stimuli in the sequence (S2, S3, S4), while in the two types of predictable sequences P1a was also reduced in response to S2 relative to S3 and S4.

**Figure 2:**
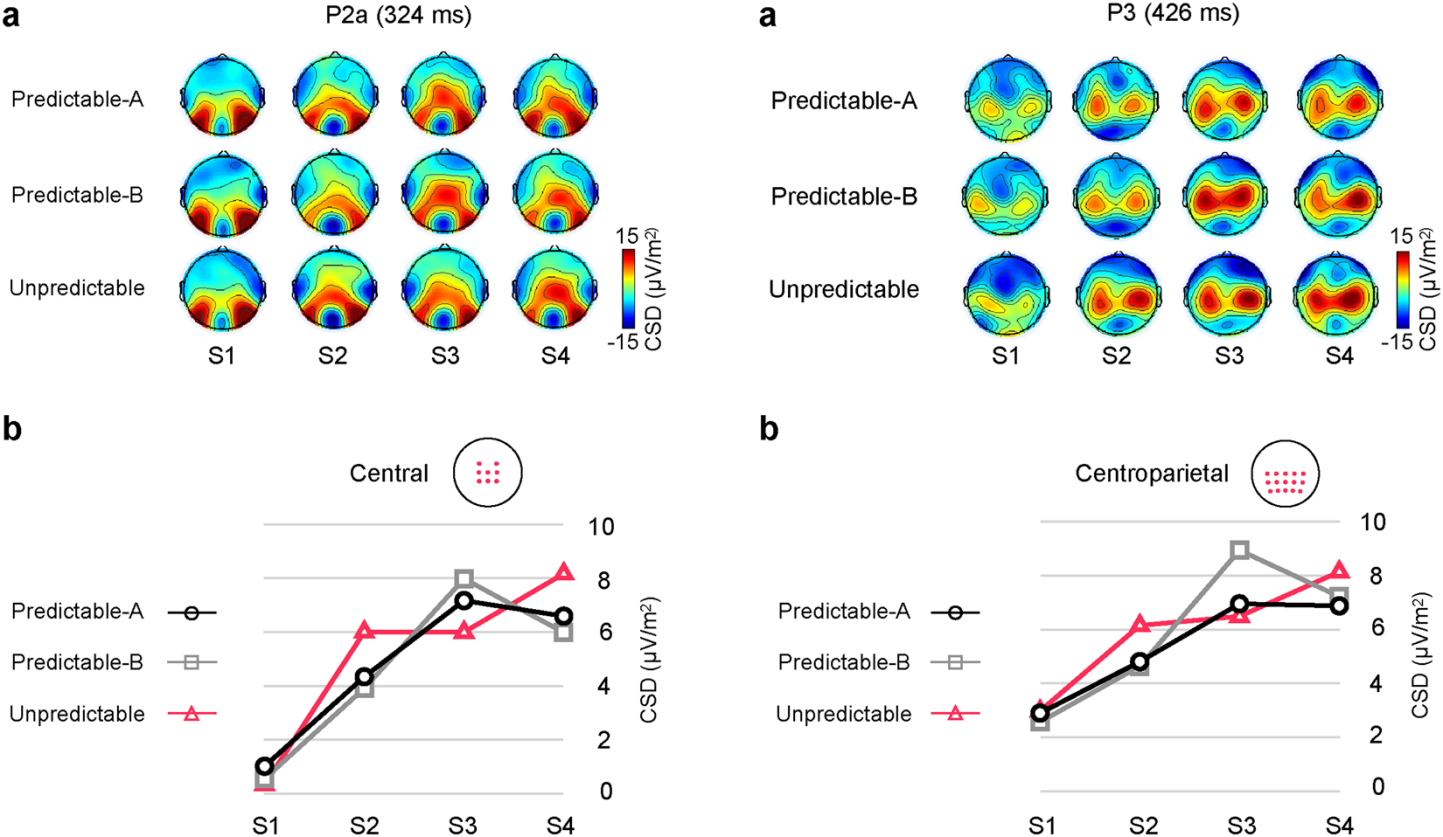
Amplitude modulations through the sequence of P2a (left panel) and P3 (right panel), measured at central and centroparietal ROIs, respectively, in each of the three conditions. (**a**) CSD distribution over the scalp for each of the four stimuli in each sequence type. (**b**) Mean amplitudes at central (P2a) and centroparietal (P3) ROIs for each stimulus in every condition (Predictable-A, Predictable-B, Unpredictable). P2a and P3 steadily increased throughout the sequences in the two predictable conditions (S1–S3), and did not differ between S3 and S4. No significant sequence-related modulations were found in the Unpredictable condition.

**Figure 3:**
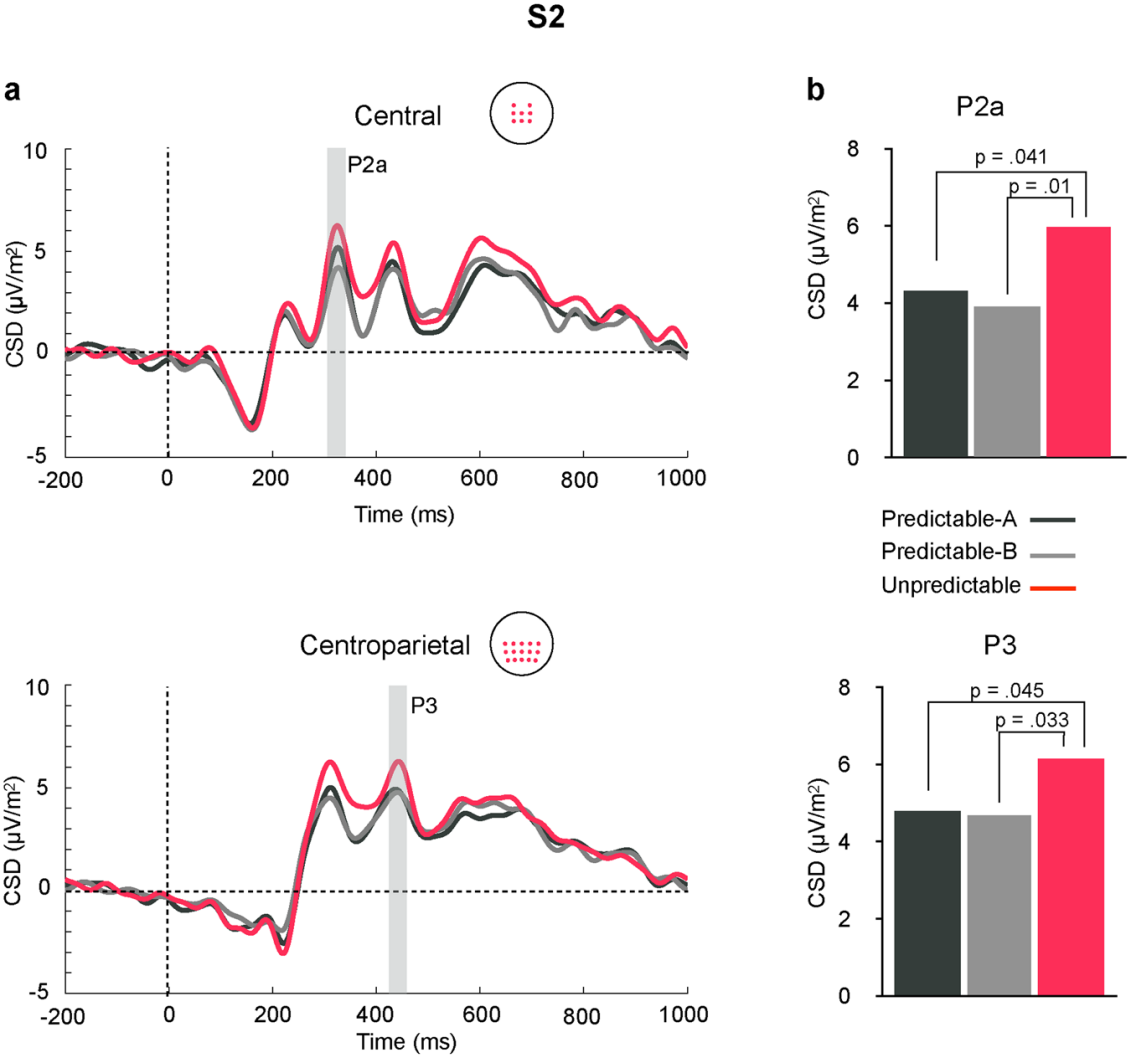
(**a**) Grand average ERP waveforms in response to S2 stimuli in each condition at the central (upper panel), and centroparietal (lower panel) ROIs. Grey bars highlight P2a and P3, where significant differences between conditions were found. Waveforms were low-pass filtered at 30 Hz for presentation purposes. (**b**) Bar plots depicting mean amplitudes of the P2a (upper panel) and P3 (lower panel) for each condition at the central and centroparietal ROIs, respectively, where these components reached their maximum. Amplitude of P2a and P3 were significantly larger in the Unpredictable than in the predictable conditions.

P2a is highlighted at central sites in Fig. 1a and b, where it peaked at 324 ms post-stimulus. A significant main effect for STIMULUS (F(3,42) = 22.34, p = 0.0001, partial η^2^ = 0.62), together with a significant two-way CONDITION × STIMULUS interaction (F(6,84) = 3.23, p = 0.007, partial η^2^ = 0.19) were found. The assumption of sphericity was not met for the CONDITION × STIMULUS interaction (Χ^2^(20) = 35.3, p = 0.022). Post-hoc comparisons indicated that in the two predictable conditions P2a amplitude progressively increased from S1 to S2 and from S2 to S3, with no further differences between S3 and S4 (Fig. 2, left panel, and Table 1). No such a progressive increase was observed in the Unpredictable condition, where the only differences consisted in P2a showing smaller amplitude in response to S1 than to all the other stimuli and larger amplitude in response to S4 than to the three preceding stimuli. In addition, this interaction showed that in response to S2 the amplitude of P2a was significantly larger in the Unpredictable than in the Predictable-A and Predictable-B conditions. Figure 3 illustrates these differences.

**Table 1:** Mean CSD values of P2a and P3 ERP components (μV/m^2^) and mean power (dB) of central theta oscillations in response to each of the four stimuli in the sequence in each condition.

	P2a				P3				Central theta			
	S1	S2	S3	S4	S1	S2	S3	S4	S1	S2	S3	S4
Predictable A	1.02	4.35	7.17	6.59	2.93	4.84	6.99	6.91	1.01	1.56	2.27	1.94
Predictable B	0.56	3.92	7.98	5.99	2.60	4.68	8.98	7.26	1.19	1.72	2.97	2.42
Unpredictable	0.34	6.01	6.00	8.17	3.01	6.19	6.73	8.18	1.21	1.94	2.04	2.29

**Figure 4:**
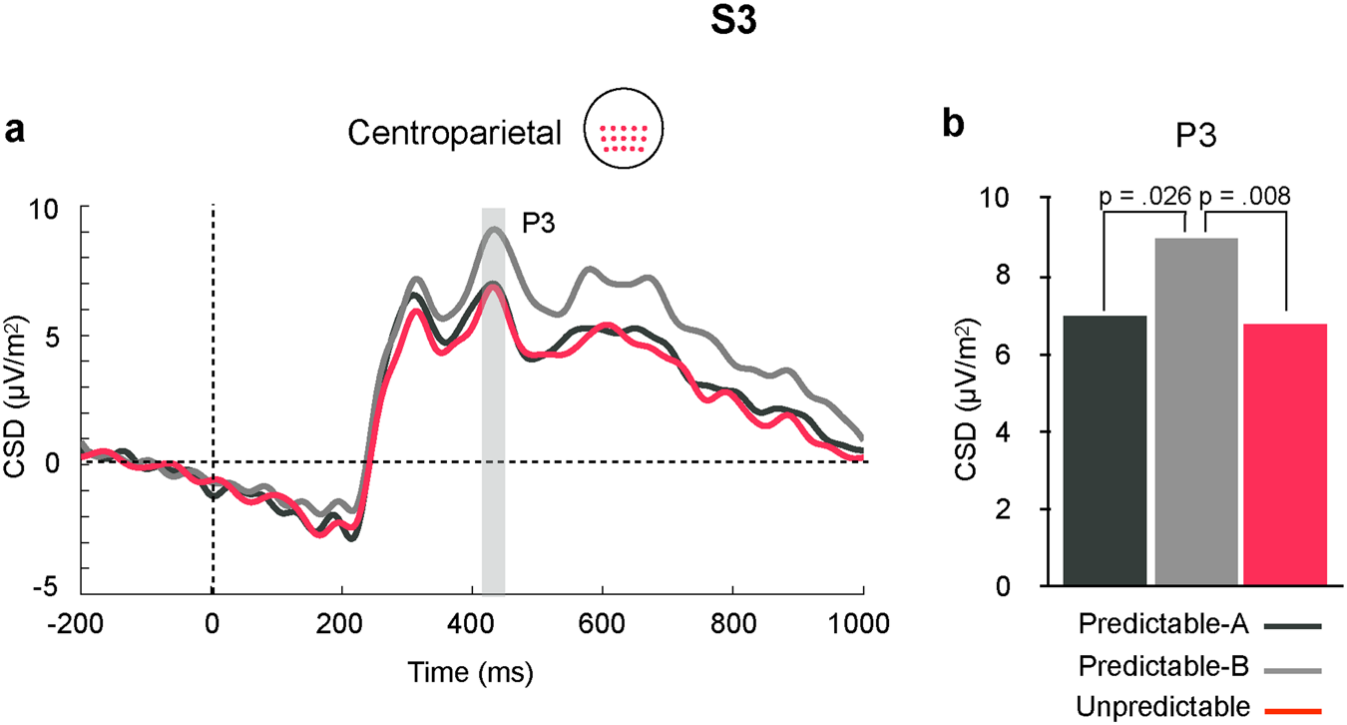
**(a**) Grand average ERP waveforms in response to S3 in each condition at the centroparietal ROI. Waveforms were low-pass filtered at 30 Hz for presentation purposes. (**b**) Bar plot depicting mean amplitude of the P3 ERP for each condition at the same ROI. Amplitude of P3 was significantly larger in the Predictable-B than in the other two conditions.

P3 is highlighted in Fig. 1 at centroparietal sites, where it peaked at 426 ms. A significant main effect for STIMULUS (F(3,42) = 19.171, p = 0.0001, partial η^2^ = 0.58), together with a significant two-way CONDITION × STIMULUS interaction (F(6,84) = 3.76, p = 0.002, partial η^2^ = 0.21), indicated that in the two predictable conditions P3 amplitude progressively increased from S1 to S2 and from S2 to S3, with no further differences between S3 and S4 (see also Fig. 2, right panel, and Table 1). No such a progressive increase was observed in the Unpredictable condition, where the only difference was that P3 amplitude was smaller in response to S1 than to all the other stimuli. Additionally, this interaction showed that, in response to S2, P3 amplitude was larger in the Unpredictable than in the two predictable conditions (see Fig. 3), while in response to S3 it was larger in the Predictable-B than in the Predictable-A and the Unpredictable conditions (see Fig. 4).

### Oscillatory activity

**Figure 5:**
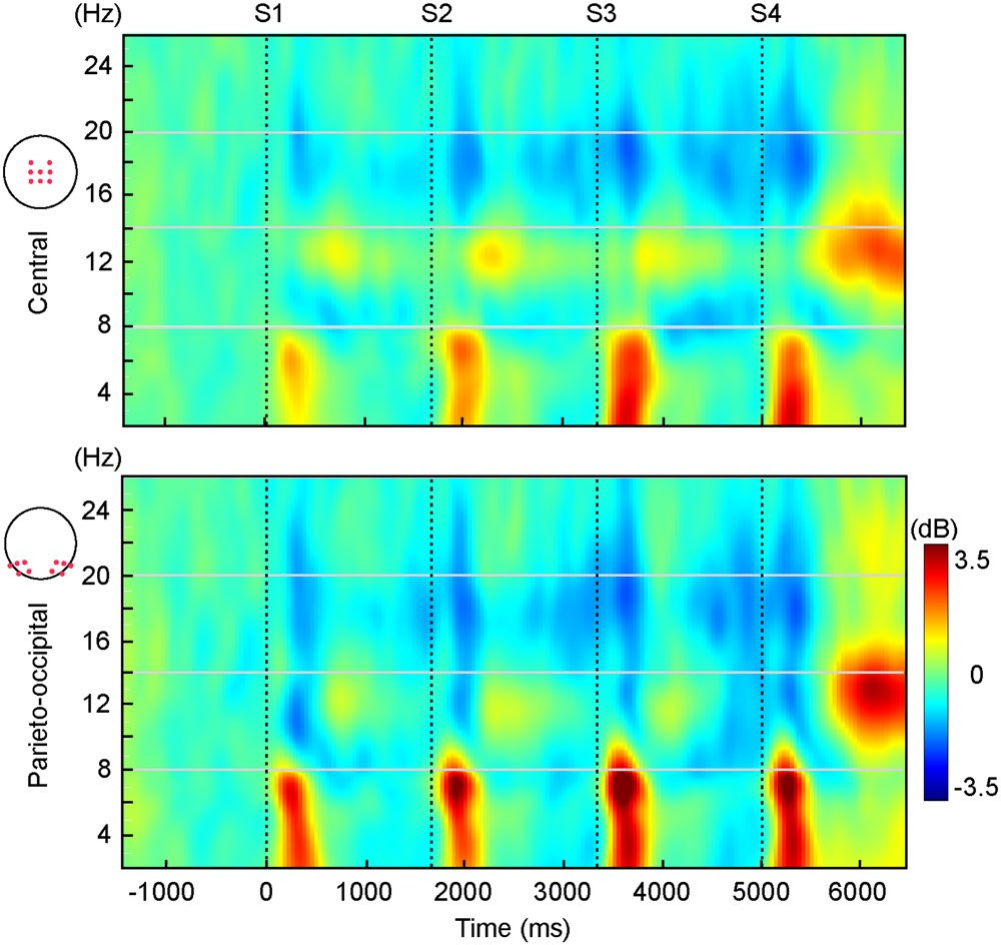
Condition-average time-frequency plot at the central and centroparietal ROIs, where analyses revealed differences between conditions in the theta and alpha bands. Figure shows TF energy averaged across single trials and baseline corrected. In the three conditions, each stimulus was associated with a transient increase in theta power (100–500 ms post-stimulus, 4–8 Hz) followed by a decrease in energy in the low-alpha band (8–10 Hz) starting about 400 ms post-stimulus and lasting until the next stimulus was presented. Activity in the high-alpha band (10–13 Hz) was associated with a power increase (500–1000 ms post-stimulus), maximal about 700 ms post-stimulus onset and lasting to next stimulus presentation. Stimulus presentation was preceded by a beta power decrease (15–24 Hz), starting in the pre-stimulus period and lasting until 400 ms post-stimulus (maximum at 300 ms post-stimulus).

Figure 5 depicts the condition-averaged TF responses associated with the presentation of the complete sequence of stimuli at a central and a parieto-occipital ROI in the theta, alpha and low-beta bands. In the three tasks, each stimulus was associated with a transient increase in theta power (100–500 ms post-stimulus, 4–8 Hz) followed by a decrease in energy in the lower part of the alpha band (8–10 Hz) starting about 400 ms post-stimulus and lasting until the next stimulus was presented. This low-alpha decrease was more pronounced in the S3-S4 interval. Activity in the higher part of the alpha range (10–13 Hz) was associated with a power increase (500–1000 ms post-stimulus), more evident at central locations, maximal about 700 ms post-stimulus onset and lasting to next stimulus presentation. Stimulus presentation was preceded by a beta power decrease (15–24 Hz), starting about 800 ms pre-stimulus and lasting until 400 ms post-stimulus (maximum at 300 ms post-stimulus).

Pre-analyses of oscillatory activity revealed differences between conditions in two different TF windows in the theta band, each with a different distribution on the scalp, and in an alpha band TF window. The first theta-band difference was observed at parieto-occipital sites in a 4–6 Hz, 120–360 ms post-stimulus TF window (parieto-occipital theta), and the second was evident at central sites in a 4–7 Hz, 280–520 ms post-stimulus TF window (central theta). Analyses of parieto-occipital and central theta were undertaken separately via 3 (CONDITION; Predictable-A, Predictable-B, Unpredictable) × 4 (STIMULUS; S1, S2, S3, S4) repeated-measures ANOVA on a parieto-ociptial and a central ROI, respectively (see Methods section). In the alpha range, differences between conditions were observed in the 200 ms period preceding S4 onset at central sites (10–12 Hz). Those differences were assessed on a central ROI via repeated-measures ANOVA with CONDITION (Predictable-A, Predictable-B, Unpredictable) as within-subject factor. For a complete description of the analyses, see the Methods section.

#### Theta band

**Figure 6:**
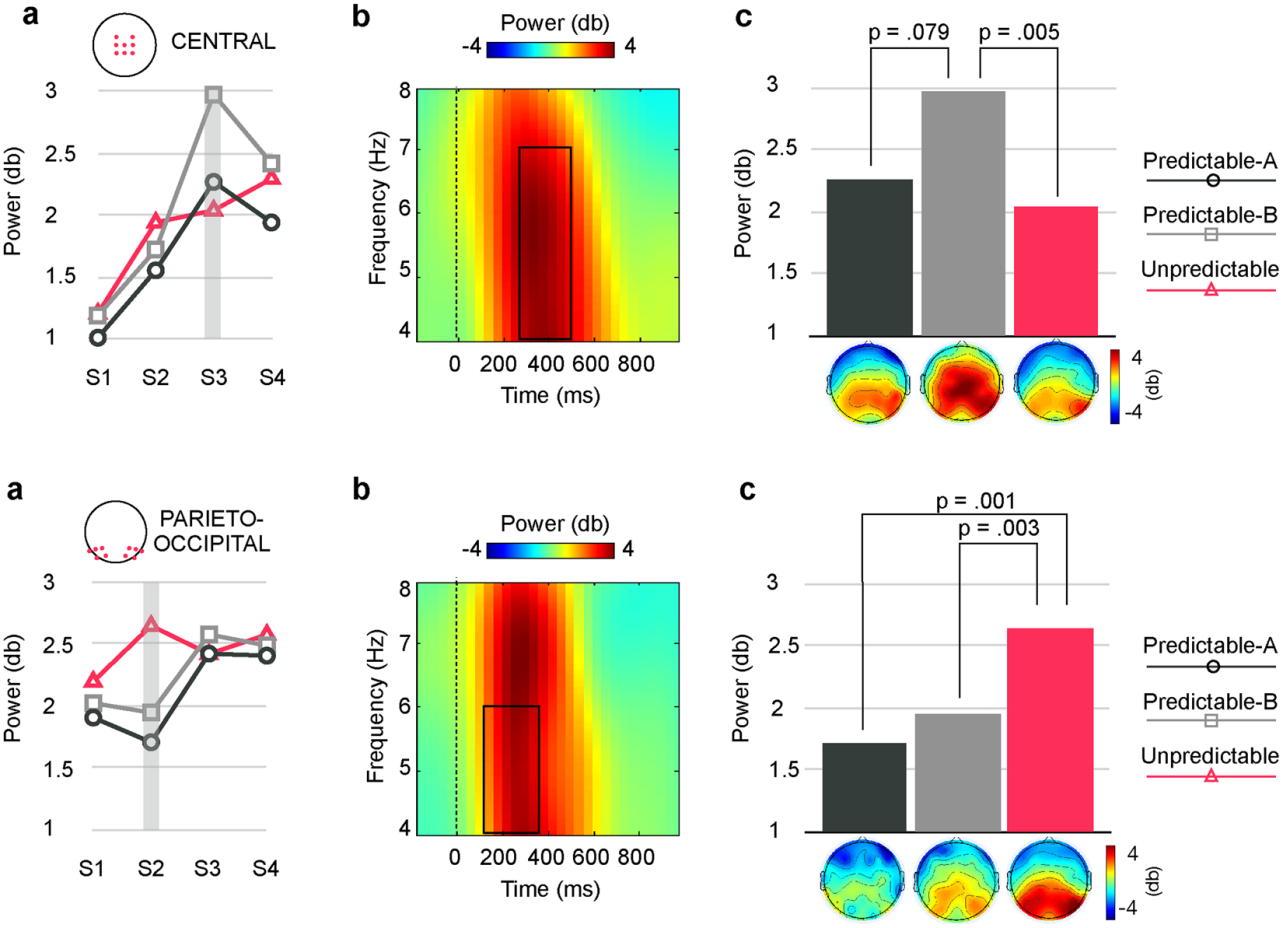
**Upper panel:** condition-average TF plots depicting post-stimulus theta power (4–8 Hz) increase at central locations. (**a**) Modulations through the sequence of central theta power in each of the three conditions. Analyses revealed differences between conditions in response to S3 (outlined in grey). Figure shows that theta power increased throughout the sequences (S1-S3). Although power decreased in response to the last stimulus in the two predictable conditions, this reduction was not statistically significant. No sequence-related modulations were significant in the Unpredictable condition. (**b**) Condition-average TF plot depicting post-stimulus theta power (4–8 Hz) relative to S3 onset at the central ROI. Dark outline indicate the TF window (280–520 ms, 4–7 Hz) where the significant differences indicated in (**a**) were found. (**c**) Bar plots depicting mean theta power in the TF window outlined in (**b**) at the central ROI. Analyses revealed significantly higher theta power in the Predictable-B than in the Unpredictable conditions. Difference between the two predictable conditions, although large, did not reach statistical significance. Topography plots showing the scalp distribution of theta power for each condition are depicted below the corresponding bar. **Lower panel:** condition-average TF plots depicting post-stimulus theta power (4–8 Hz) increase at lateral parieto-occipital locations. (**a**) Modulations through the sequence of central theta power in each of the three conditions. Analyses revealed differences between conditions in response to S2 (outlined in grey). No sequence-related modulations were found for any condition. (**b**) Condition-average TF plot depicting post-stimulus theta power (4–8 Hz) relative to S3 onset at the parieto-occipital ROI. Dark outline indicate the TF window (120–360 ms, 4–6 Hz) where the significant differences indicated in (**a**) were found. (**c**) Bar plots depicting mean theta power in the TF window outlined in (**b**) at the central ROI. Analyses revealed significantly higher theta power in the Unpredictable than in the two predictable conditions.

Figure 6 depicts the results obtained in the analyses of central (upper panel) and parieto-occipital (lower panel) post-stimulus theta enhancements.

Central Theta. Significant main effects for CONDITION (F(2,28) = 3.37, p = 0.049, partial η^2^ = 0.19) and STIMULUS (F(3,42) = 21.29, p = 0.0001, partial η^2^ = 0.60), and a significant two-way CONDITION × STIMULUS interaction (F(6,84) = 2.88, p = 0.046, partial η^2^ = 0.17) were found. The assumption of sphericity was not met for the main effect of STIMULUS (Χ^2^(5) = 15.55, p = 0.008) and for the CONDITION × STIMULUS interaction (Χ^2^(20) = 45.47, p = 0.001). Post-hoc analyses indicated that in the two predictable conditions central theta progressively increased over the sequence, from S1 to S2 and then to S3, with no further differences between S3 and S4, while in the Unpredictable condition no such a steady increase was observed, the only difference throughout the sequence being theta power showing significantly lower power in response to S1 than to all the other stimuli in the sequence (see also Table 1). Analyses also revealed that central theta power differed between conditions in response to S3, where it was significantly higher in the Predictable-B than in the Unpredictable condition.

Parieto-occipital Theta. Significant main effects for CONDITION (F(2,28) = 3.54, p = 0.043, partial η^2^ = 0.19) and STIMULUS (F(3,42) = 5.43, p = 0.003, partial η^2^ = 0.28) and a significant two-way CONDITION × STIMULUS interaction (F(6,84) = 4.90, p = 0.0001, partial η^2^ = 0.26) were found. The assumption of sphericity was not met for the main effect of STIMULUS (Χ^2^(5) = 16.56, p = 0.006). Post-hoc analyses indicated, on the one hand, that in the Unpredictable condition power was significantly lower in response to S1 than to the other stimuli in the sequence, with no further differences between these latter, while in the two predictable sequences power did not differ between S1 and S2 but was in both cases significantly lower than in response to S3 and S4, which did not differ between them. On the other hand, analyses revealed that parieto-occipital theta power differed between conditions in response to S2, where it was significantly higher in the Unpredictable than in the Predictable-A and Predictable-B conditions.

#### Alpha/Beta bands

**Figure 7:**
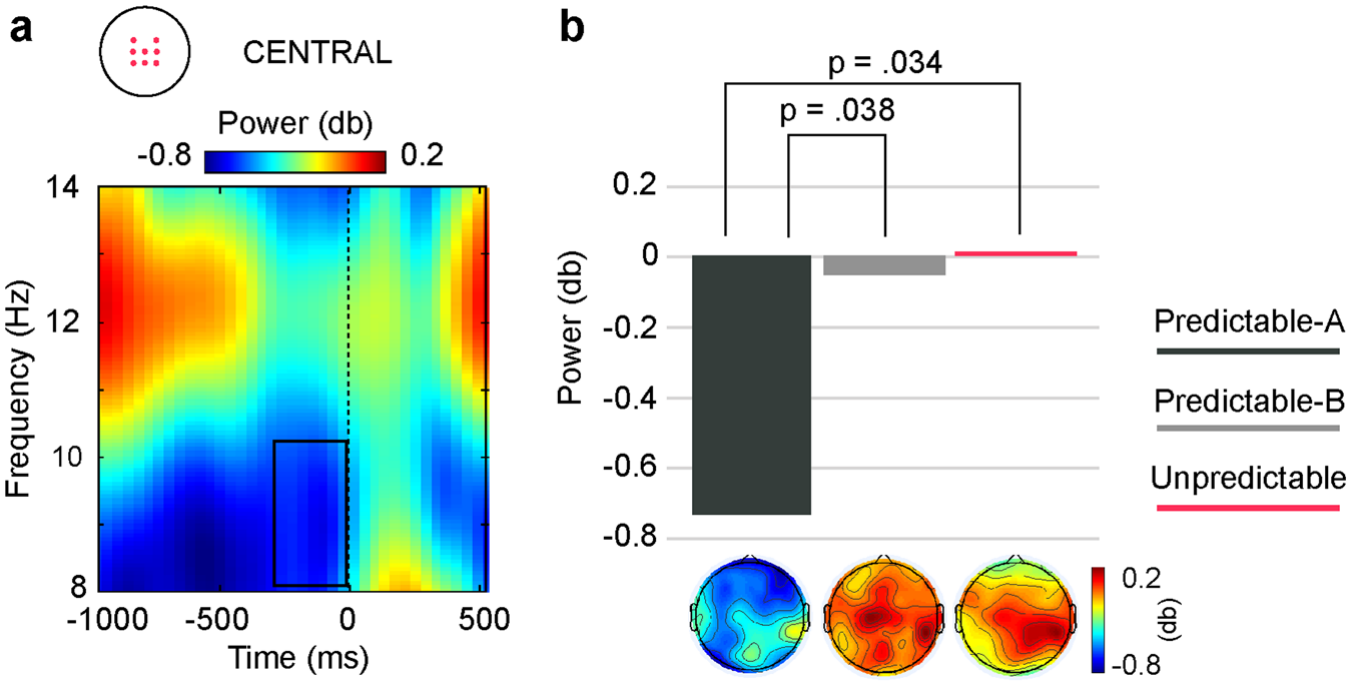
(**a**) Condition-average TF plot relative to S4 onset at the central ROI. Dark outlines indicate significant TF window, corrected for multiple comparisons (false discovery rate, FDR, p < 0.05), where significant differences between conditions were found. (**b**) Bar plots depicting average alpha power for that TF in each condition. Alpha power decrease preceding S4 onset was significantly deeper in the Predictable-A than in the two other conditions. Topography plots showing the scalp distribution of alpha power decrease for each condition are depicted below the corresponding bar.

As illustrated in Fig. 7, a significant main effect for CONDITION (F(2,28) = 3.42, p = 0.047, partial η^2^ = 0.20) was found, indicating that the alpha power decrease preceding the last stimulus of the sequence (S4) was much more marked in the Predictable-A than in the Predictable-B and Unpredictable conditions in the last 200 ms pre-S4 onset.

## Discussion

We investigated prediction build-up trough evidence accumulation over time by presenting participants with unpredictable and increasingly predictable four-stimuli sequences. Compared to unpredictable series, predictable sequences were associated with a steady increase of P2a and P3 amplitudes as well as of oscillatory theta power, with a deeper suppression of alpha power in anticipation for the final stimulus, and with an initial decrease and later rebound of P1a amplitude. Progressive P2a and P3 amplitudes, and centrally distributed theta band power increases would reflect the accumulation of evidence throughout the sequence. This evidence accumulation would contribute to sharpen predictions over time, leading to correctly predicting the last stimulus in the sequence, as reflected by the marked alpha power decrease preceding that stimulus in the Predictable-A condition. Unlike P2a, P3 and theta oscillations, alpha activity was not sensitive to the progressive accumulation of evidence, but indexed participants’ certainty about an upcoming stimulus resulting from the information gathering process. Finally, P2a, P3 and central theta, as well as parieto-occipital theta oscillations, were sensitive to discrete variations in PE associated to the stimuli.

In the predictable sequences, P2a amplitude progressively increased between S1 and S3. This is in contrast with what was observed in the unpredictable series, in which no such increment was found. This result parallels our estimations about the accumulation of predictive information from each stimulus to the next in the predictable conditions, which also increases steadily through the sequences (see Tables 1 and 2), while in the Unpredictable sequences remains relatively constant. In the predictable sequences, predictive information in anticipation for S4 is maximal and that last stimulus is fully predictable. Accordingly, no further amplitude increase was observed between S3 and S4 in those series. This result points to the possible role of P2a in evidence accumulation for prediction build-up. Furthermore, it is coherent with previous studies in which positive components with similar latencies and anterior scalp distributions were related to evidence accumulation processes^10,12^. In those works, a frontally distributed component peaking about 350 ms post-stimulus, labelled prefrontal P2 (pP2), was proposed to be associated with evidence accumulation to reach decisions^10–12^. In the present experiment, P2a arises in the same latency range as the pP2 described in those studies, although its distribution is not as frontopolar, reaching its maximum at central and frontocentral sites. Components in the latency range of P2 have been labelled differently in the various paradigms in which they have been described and have been reported to show slightly different anterior topographies, from frontopolar to frontal-central^10^, probably depending on the particularities of the experimental design and task demands. These components have been associated to different processes, such as attentive and feature-based stimulus evaluation^39^, the recall of task rules^40^ or the activation of stimulus-response (S-R) associations^41^, mostly related to the evaluation of a stimulus to select an adequate response. Even when described in relation to evidence accumulation, it has been suggested that this component might be associated to the activation of S-R representations to reach a decision about the appropriate response^10^. The steady increase of P2a amplitude observed in the present experiment is compatible both with the evaluation of stimuli’s features, whose relevance progressively increase throughout the sequence, and with the accumulation of information over the predictable sequences in order to more accurately anticipate upcoming stimuli. However, in our experiment no response was required, which would indicate that while response-related processing may contribute to modulate the amplitude of this component, at least a part of it would be response-independent. P2a would therefore reflect a processing stage associated to the extraction and accumulation of feature-based stimulus information.

**Table 2:** Summary of marginal (e_t_ = i) and conditional (e_t_ = i|e_t-1_ = j, e_t_ = i|e_t-1_ = j, e_t-2_ = k, e_t_ = i|e_t-1_ = j, e_t-2_ = k, e_t-3_ = l, considering the preceding, two preceding and three preceding events, respectively) probabilities for each stimulus in the sequence in each condition (prob), along with the accumulated predictive information (p) on the forthcoming stimulus at each sequence position.

		Predictable-A		Predictable-B		Unpredictable	
		prob	p	prob	p	prob	p
S1	(e_t_ = i)	0.25	—	0.25	—	0.25	—
S2	(e_t_ = i)	0.25	—	0.25	—	0.25	—
	(e_t_ = i|e_t-1_ = j)	0.33	0.40	0.33	0.40	0.17	−0.56
S3	(e_t_ = i)	0.25	—	0.25	—	0.25	—
	(e_t_ = i|e_t-1_ = j)	0.5	1	0.5	1	0.33	0.40
	(e_t_ = i|e_t-1_ = j, e_t-2_ = k)	0.5	1	0.5	1	0.33	0.40
S4	(e_t_ = i)	0.25	—	0.25	—	0.25	—
	(e_t_ = i|e_t-1_ = j)	0.5	1	0.5	1	0.33	0.40
	(e_t_ = i|e_t-1_ = j, e_t-2_ = k)	1	2	1	2	0.33	0.40
	(e_t_ = i|e_t-1_ = j, e_t-2_ = k, e_t-3_ = l)	1	2	1	2	0.33	0.40

Table shows, when appropriate, the accumulated information (bits) due to the knowledge of the preceding stimulus (e_t_ = i|e_t-1_ = j), the last two stimuli (e_t_ = i|e_t-1_ = j, e_t-2_ = k), and last three stimuli (e_t_ = i|e_t-1_ = j, e_t-2_ = k, e_t-3_ = l) for each position in the sequence. Marginal probability of any stimulus was the same for every position in the sequence (0.25). Conditional probabilities at each sequence position differed between conditions.

The progressive increase of P3 amplitude between S1 and S3 in the predictable sequences fits the classical relation of P3 to context updating^15^ and confidence encoding^17^. Moreover, as P2a, it parallels our estimates about predictive information accumulation (see Tables 1 and 2). In a decision-making experiment, O’Connell and colleagues^19^ identified a centro-parietal positivity (CPP) that increased with incoming evidence and that shared all of the characteristics of the P3 component^20^. They proposed that it reflected domain-general processes in relation to decision build-up, independently of the specific content of sensory and motor processing^19^. We believe our results are coherent with the idea of this component indexing the existence of such a domain-general, and perhaps purpose-general, evidence-accumulation mechanism in the brain, supporting in this experiment prediction build-up over time. In this regard, it is important to keep in mind that participants were not informed about the existence of unpredictable sequences, which excludes the possibility that the lack of significant P3 modulations in the unpredictable condition was due to subjects knowing from early in the sequence that correctly predicting upcoming stimuli was not possible. Rather, from their perspective predictable and unpredictable series would have been perceived as “easy” and “difficult” versions of the task. Actually, P3 also showed some progressive amplitude increase in the unpredictable sequences (Figs 1 and 2) that, although non-significant, might reflect participants’ unsuccessful effort to gather information in those series as well. Interestingly, while this non-significant P3 increase in the Unpredictable condition continued throughout all the sequence (S1–S4), in the predictable conditions it was observed only between S1 and S3, that is, until enough information to accurately predict the upcoming stimulus was accumulated, but not after that moment, which reinforces the interpretation of this component as reflecting an information accumulation process supporting prediction build-up. Finally, it is worthy to note, first, that differently to those previous works in which similar increases of P3 amplitude were observed, no overt response was required in our experiment, which excludes the involvement of response-related processes in the observed P3 amplitude effect and, second, that our experiment did not involve making perceptual decisions about the presence of a target stimulus, but anticipating the identity of a stimulus yet to come on the basis of the probabilities extracted from the information gathered at each particular moment, which gives support to the notion of predictions represented as probability distributions, as suggested by predictive coding models.

Regarding oscillatory activity, in the present experiment two different theta power components were found, one distributed over parieto-occipital regions and another broadly spread over central locations, the former reaching its maximum power 30–40 milliseconds before the latter. Increasing predictability through information accumulation was related to post-stimulus centrally distributed theta power steadily increasing between S1 and S3, with no further differences between S3 and S4. Two groups of results have been described in relation to theta activity. On the one hand, theta power has been shown to increase over frontocentral regions as more information is gathered in experimental contexts such as perceptual decision making^23^, in which theta activity has been identified as an index of evidence accumulation^24^. Our results are coherent with this claim, and the presumable role of theta activity in evidence accumulation is supported by the fact that its increase paralleled both the progressive P3 amplitude increase described above and our estimates about the predictive information available to anticipate an upcoming stimulus at each time point (see Tables 1 and 2). Furthermore, it is coherent with the well-established relationship between P3 and theta oscillations^42–44^ and with the notion of theta activity playing a general role in integrating information from diverse cognitive systems for different purposes^27,28^. On the other hand, theta has been linked to comparing incoming with expected events^45–47^, and has been shown to be modulated by the degree of discrepancy between expected and actual events^45^, that is, by PE. Actually, it has been proposed as a marker of the utilization of PE information by the executive-control systems in the brain^37,45,48^. Given the relation between theta oscillations and PE, we might speculate that theta activity could constitute the flipside of P3 modulations in relation to evidence accumulation, with P3 reflecting the accumulation of evidence provided by the successive stimuli and theta oscillations indexing the gathering of PE information for updating and sharpening predictions. However, our design does not allow us to independently and systematically manipulate PE and the amount of predictive information available for prediction, and thus to disentangle the possible differential contributions of both sources of information to prediction build-up through evidence accumulation and their electrophysiological correlates. Further research must be conducted to clarify this point.

Along with central theta, a strong theta power increase was observed within the first 300 ms post-stimulus over posterior recording locations, probably reflecting processes related to stimulus encoding^24,49^. Interestingly, these posterior theta oscillations were not modulated throughout the sequence and thus did not seem sensitive to evidence accumulation, but showed significantly higher power in the unpredictable than in the two predictable conditions in response to S2. The fact that the probability of S2 being part of an unpredictable sequence was much lower than that of it belonging to a predictable sequence points to the relation of this posterior theta to PE, as is discussed below.

As the culmination of the evidence accumulation process, significantly larger low-alpha power suppression was observed immediately preceding the onset of the final stimulus under predictable conditions. Alpha power decrease has been described preceding events that can be anticipated^50–52^. Therefore, it would indicate here that enough evidence for making an accurate prediction has been accumulated over the first three stimuli. The fact that P3 amplitude and central theta power did not further increase in response to the last stimulus supports this interpretation, since it would indicate that S4 was predictable and no more information was needed.

Finally, P1a amplitude was reduced in response to S2 in comparison to S3 and S4 in the predictable sequences. In the context of decision-making, a positive anterior component between 150–250 ms has ben associated to perceptual stimulus evaluation^10^. Similarly, in prediction research positive components in the range of 190–250 ms have been related to expectancy driven by stimulus probabilities and are thought to reflect a neural process of comparison between sensory inputs and internal predictions, i.e., PE^32,53,54^. In other words, the more surprising events are, the largest the amplitudes of these components will be. According to this notion, the attenuation of S2-locked P1a in the predictable sequences observed here would reflect PE reduction. One would expect that P1a decrease would become larger throughout the rest of the sequence, as evidence accumulates and PE reduces. However, this decrease was instead followed by an amplitude increase, in agreement with previous results where similar rebounds in PE-related activity were observed in series of repetitive^6^ and regular, predictable stimuli^4^. In those experiments, the first stimulus completely predicted the rest of the sequence, which led authors to hypothesize that the rebound effect could result from the increased confidence in predictions sharpening neural anticipatory activity around the preferred prediction, leading to paradoxical PE increase insofar as predictions are rarely absolutely precise^4^. Our results extend their findings in that in our task observers needed to update their predictions as information accumulated after each new stimulus, given that uncertainty steadily decreased as more items were presented and only the last stimulus in the predictable series became fully predictable.

Our protocol also allowed us to investigate PEs of mispredicted and unpredicted stimuli. According to Arnal and Giraud^55^, mispredicted stimuli are associated with larger PE because they induce both error generated by the pre-activated representations that do not match the sensory input and by the activation elicited by sensory input that is not anticipated, while unpredicted stimuli induce only the latter type of error. Recently, Hsu and colleagues^8^ found evidence for this notion. They observed larger PE-related ERP activity for mispredicted than for predicted and unpredicted auditory stimuli, and also larger for predicted than for unpredicted. Note that according to predictive coding models even predicted stimuli are associated with some amount of PE, which would explain that pattern of results. In our experiment, however, only the last event in the predictable sequences could be predicted. All the other stimuli were associated to some degree of uncertainty (surprise), progressively decreasing in the predictable series and relatively constant in the unpredictable ones. This allowed us to extend our analyses to the preceding stimuli to further investigate into how PE is implemented. Assuming that predictions are represented as probability distributions^56^, in a context where there is limited number of possible stimuli the difference between mispredicted and unpredicted stimuli may rest on the perceived probability of the predicted events to occur and/or on the information available to anticipate their occurrence, so that mispredicted and unpredicted stimuli could be regarded as violations of high and low probability predictions, respectively, within that distribution. In other words, they would constitute more or less surprising events over a continuum of predictions, which would be reflected in PE size. Coherently with our information estimates, in response to S2 we observed significantly higher parieto-occipital theta power in the unpredictable than in the predictable sequences. As stated above, theta activity has been linked to comparing incoming with expected events^37,45–47^, and has been shown to be modulated by the degree of discrepancy between them, that is, by PE. Theta power increase over parieto-occipital regions has been related, for example, to the visual mismatch negativity (vMMN)^57^, an ERP component widely considered as a perceptual PE signal^1,6,58,59^, and the increase observed here could be thus taken as an indicative of higher PE elicited by S2 in the unpredictable condition. Furthermore, P2a and P3 amplitudes were also enhanced. Although the literature on the role of P2 in predictive contexts is sparse, given its aforementioned association with information accumulation it is possible that the large amplitude increase observed was related to the fact the S2 was highly informative in the unpredictable sequences. P3 amplitude enhancement would also be coherent with this explanation; the highly informative S2 would prompt participants to re-evaluate their predictions, in agreement with previous findings linking P3 amplitude to the degree of surprise carried the eliciting stimulus^16^, and to the revision of expectations about the current task context^60^.

In response to S3, however, P3 amplitude and central theta power were enhanced in response to stimuli changing rotation direction (Predictable-B) with regards to the Unpredictable and the Predictable-A conditions, although the difference in theta power between the two predictable conditions, albeit large, did not reach statistical significance. No effects in either P2a amplitude or posterior theta power were observed. This result was unexpected, since S3 was preceded by the same amount of predictive information in both Predictable-A and Predictable-B sequences and both stimuli were equally probable or surprising. We hypothesize that rotation change in S3 was nevertheless surprising for the participants and that a misprediction occurred. The violated prediction would be, however, based on non-probabilistic criteria or on probabilities of higher order not intrinsic to the task, but given by general experience in the environment and not taken into account for our estimations. Thus, one could think, for instance, that in our real world it is more “natural” to expect a rotation to continue in the same direction than to go backwards once it started rotating in a particular direction. Such an explanation needs, however, from further systematic research to be tested.

In sum, we identified electrophysiological correlates of evidence accumulation for prediction build-up, and showed how these correlates are also sensitive to discrete variations in the amount of information provided by a stimulus. These results are in agreement with the notion of predictions represented as probability distributions continuously fine-tuned over time. However, they suggest that probability alone cannot account for the way predictions are implemented in the brain and indicate, first, that mispredictions can occur regardless of the objective predictability of events, affecting prediction build-up over time and, second, that PE presumably require different processing resources depending on whether they result from unpredicted or mispredicted events.

## Materials and Methods

### Participants

15 participants (11 female, 24.9 ± 2.7 years) took part in the study and received monetary compensation for their participation. All participants were right-handed students with normal or corrected-to-normal vision and reported no history of neurological or psychiatric disorders. Informed consent was obtained from them. Experimental procedures were undertaken in accordance with the Declaration of Helsinki and with the approval by the Comité de Protection des Personnes Ile de France II.

### Stimuli and procedures

**Figure 8:**
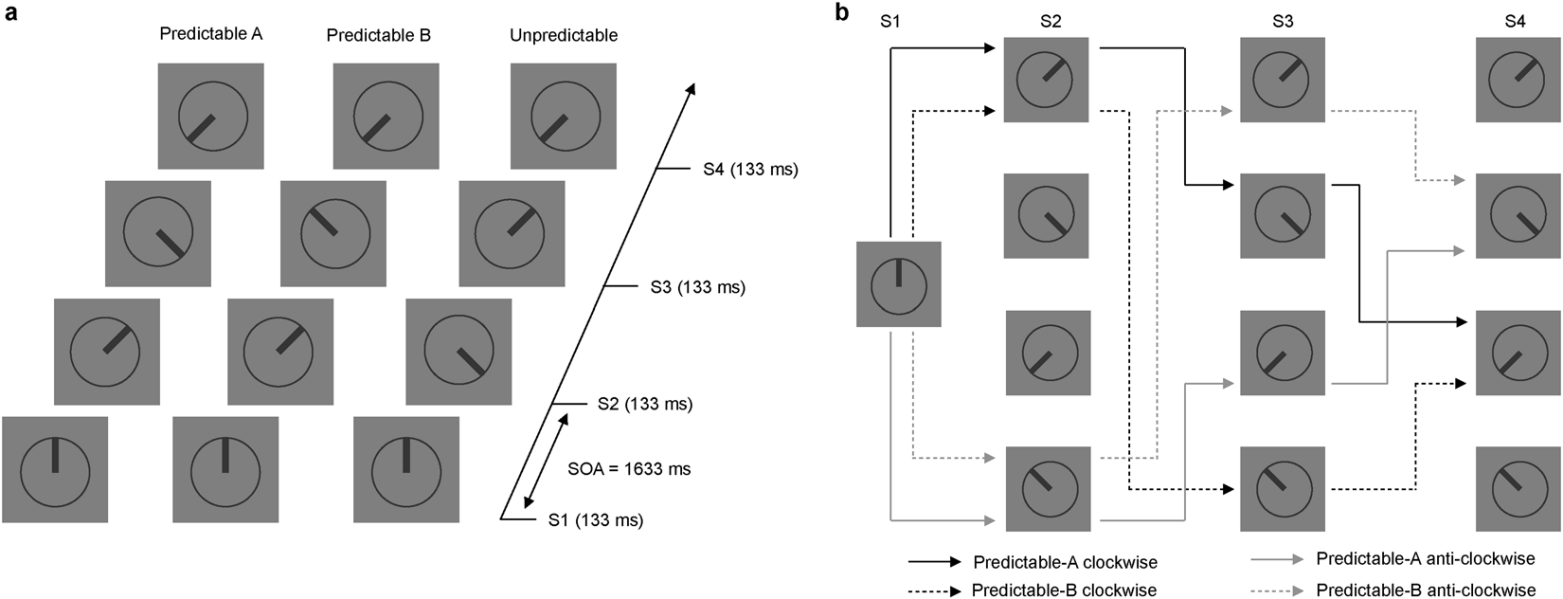
(**a**) Schematic representation of the task, indicating the timing of the sequences and three possible sequences corresponding each to the Predictable-A, Predictable-B and Unpredictable conditions The initial position (S1) could be 12, 3, 6 or 9 in the clock. In the predictable sequences, the hand in the clock could rotate either clockwise or anti-clockwise, the hand pointing to intermediate positions between 12, 3, 6 and 9 in the clock. In 50% of these predictable sequences the rotation direction did not change throughout the sequence (Predictable-A), but in 50% of them there was a rotation change in S3 (Predictable-B). In 66% of the sequences the last stimulus (S4) was fully predictable after S3 (Predictable-A and Predictable-B), and in 33% it was unpredictable. Every stimulus was presented for 133 ms, with 1500 ms ISI (1633 SOA). Inter-sequences interval was set on 3000 ms. (**b**) Diagram representing all the possible stimuli sequences in the Predictable-A and Predictable-B conditions for a given initial stimulus (12 in the clock). Sequences could initially (S2) rotate either in a clockwise (black lines) or anti-clockwise (grey lines) manner. In S3, however, rotation could follow the same rotation direction indicated by S1 and S2 (continuous lines, Predictable-A condition), or change rotation direction from clockwise to anti-clockwise or vice versa (dashed lines, Predictable-B condition). S4 position was fully determined by the positions of S2 and S3 in both types of sequences.

Visual stimuli were presented on a 27-in. 60 Hz LCD monitor. Stimuli were presented against a grey background at the centre of the screen and consisted of displays containing a clock-like circle (2.85° of visual angle) with a thick line inside pointing in one of eight possible directions, as illustrated in Fig. 8(a). On each trial, four displays (S1-S2-S3-S4) were presented successively for 133 ms each and separated by a blank interval of 1500 ms. Each sequence started with the line pointing 12, 3, 6 or 9 hours. In S2, S3, and S4 stimuli could point to any of four intermediate positions between those (approximately 2, 5, 7, or 10 on the clock). No stimulus in any condition had the same orientation than the preceding one. Unbeknownst to participants, from S2 onwards three different types of sequences were possible: S2 could rotate 45° either clockwise or anti-clockwise with respect to S1 (predictable sequences) or point to any of the two other possible positions (unpredictable sequences). Predictable sequences developed according to the following rules: first, clockwise or anti-clockwise S1-S2 rotations were equally probable; second, S3 orientation could follow the rotation direction indicated by S1-S2 (Predictable-A condition), or change rotation direction from clockwise to anti-clockwise or vice versa (Predictable-B condition), with Predictable-A and Predictable-B conditions being equally probable; finally, in both predictable conditions S4 followed the rotation direction indicated by S2-S3 so that S4 orientation was fully predictable. Figure 8(b) depicts an example diagram of all the possible Predictable-A and Predictable-B sequences for a given S1 stimulus. In the Unpredictable condition, S3 and S4 pointed in random directions, so that positions were not predictable, with the only restriction that if there was a S2-S3 45° rotation, S4 could not point in a direction that could be perceived as continuing that rotation.

Series corresponding to each condition (Predictable-A, Predictable-B, Unpredictable) were equally probable and randomly intermingled (0.33 probability each). Marginal probability of each orientation at each sequence position, i.e., its probability of occurring, not conditioned on preceding events, was 0.25 in every condition, so that participants had to rely on their knowledge about the preceding events and about the conditional probabilities between them in order to predict the forthcoming stimuli. Importantly, rather than memorizing sequences of events, participants had to base their predictions on the implicit rotation rules. Table 2 contains a complete description of marginal and conditional probabilities, associated to each sequence position in each condition, resulting from this design.

Participants were given written instructions to maintain their gaze on the central fixation cross and to predict at every point what the next stimulus in a sequence would be. Participants were not required to give any response regarding their predictions. This approach has proven to be effective in generating prediction-related brain responses in previous studies^8,32^. However, to ensure that subjects were focused on the task they were required to press a key anytime a single stimulus was identical to that presented immediately before in that sequence (catch trials). These constituted 5% of the trials and were not considered for analysis. Before data recording, a block of 80 trials was administered to allow participants to familiarize with the task. E-prime version 2.0 (Psychology Software Tools) was used for presenting the stimuli.

### Information estimates

Within the framework of Shannon’s information theory^34^, we followed previous studies^30^ for computing surprise and predictive information on the forthcoming stimulus at each time point in the predictable and unpredictable series to quantify PE and prediction build-up through evidence accumulation. Calculations are detailed in the supplementary materials. Within that theory, surprise is a measure of the improbability of an event, and is defined by the marginal probability of that event to occur, while predictive information quantifies the amount of information available to predict an upcoming stimulus, i.e., surprise reduction, and is defined by conditional probabilities^30^. Marginal probability reflects the probability of an event occurring, not conditioned on preceding events. Conditional probability, on the other hand, reflects the probability of an event to occur given that certain precedent events occurred. Table 2 summarizes marginal and conditional probabilities and accumulated predictive information estimates. Predictive information steadily accumulated throughout the sequence in the Predictable conditions (0.41, 1 and 2 bits for S2, S3 and S4, respectively), increasing predictability of forthcoming stimuli on the basis of knowledge about preceding events. In the Unpredictable sequences, however, the amount of predictive information remained low and relatively constant (−0.58, 0.41, 0.41 bits for S2, S3 and S4, respectively).

Table 3 contains estimates of surprise elicited by stimuli at each sequence position in each condition. Calculations are based on conditional probabilities. With the obvious exception of S1, surprise elicited at every sequence position was larger in the Unpredictable than in the Predictable conditions, and was particularly large for S2.

**Table 3:** Surprise (bits) elicited by stimuli at each sequence position in each condition.

	S1	S2	S3	S4
Predictable-A	2	1.6	1	0
Predictable-B	2	1.6	1	0
Unpredictable	2	2.56	1.6	1.6

### Electrophysiological recordings

Continuous EEG data (0.1–250 Hz band-pass) were collected from 60 actiCAP EEG electrodes (BrainProducts GmbH) mounted on an elastic cap. EEG electrodes were placed following the extended 10–10 position system (Fp1, Fp2, AF7, AF3, AF4, AF8, F7, F5, F3, F1, Fz, F2, F4, F6, F8, FT7, FC5, FC3, FC1, FC2, FC4, FC6, FT8, T7, C5, C3, C1, Cz, C2, C4, C6, T8, TP7, CP5, CP3, CP1, CPz, CP2, CP4, CP6, TP8, P7, P5, P3, P1, Pz, P2, P4, P6, P8, PO7, PO3, POz, PO4, PO8, O1, Oz, O2) and were referenced to right mastoid. Four additional electrodes were placed above and below the left eye and on the outer canthi of both eyes to monitor blinks and eye movements. EEG and EOG data were collected using the PyCorder system and actiCHamp amplifiers (BrainProducts GmbH, Gilching, Germany) in DC recording mode with a sampling rate of 2000 Hz.

### EEG analyses

EEG data were processed using EEGLAB^61^ (Swartz Center for Computational Neurosciences, La Jolla, CA: http://www.sccn.ucsd.edu/eeglab) running under MATLAB R2012b (Mathworks, Navick, MA). Pre-processing was performed as follows. EEG data were re-referenced offline to linked mastoids. Bad channels were then identified by visual inspection and excluded from processing. Epochs for each stimulus type were extracted from −2000 to +7000 ms with respect to the first stimulus in each sequence, and were inspected for non-stereotyped artifacts and removed if present (9.55% ± 3.99 of trials removed). Stereotyped artifacts, including blinks, eye movements and muscle artifacts were deleted via independent component analysis (ICA) using the extended infomax algorithm^62^. The average number of independent components removed was 3.33 (±0.98 SD). The remaining components were then projected back into electrode space. After ICA, channels that were deemed bad were reintroduced by interpolating data between neighbouring electrodes using spherical spline interpolation^63^. The average number of trials per condition was 96.91 (±9.41 SD). Finally, EEG data were transformed using a surface Laplacian filter (smoothing = 10^−5^, number of iterations = 10, spherical spline order = 4) to reduce volume conduction effects in EEG electrode space (CSD Toolbox^64^).

#### Event-related Potentials Analyses

ERP analyses were performed on ICA-corrected epochs time-locked to the onset of each stimulus (−200 to +1200 ms). To minimize the influence of individual differences in topographies as well as the effects of performing multiple statistical comparisons, the analyses of the ERP components and significant oscillations under study were performed on different ROIs of relevant sites, selected on the basis of both the grand average visual detection of the maximal peak electrodes and the topographical distribution of the activity on the scalp (see Fig. 1). Following this procedure, P2a and P3 were respectively measured on frontocentral (including F1, Fz, F2, FC1, FC2, C1, Cz, C2), central (including FC1, FC2, C1, Cz, C2, CP1, CPz, CP2), and centroparietal (including C1, Cz, C2, CP1, CPz, CP2, P1, Pz, P2) ROIs in a 20 ms window with regards to the most positive point in the latency range of 215–230 ms, 315–330 ms and 415–430 ms for P1a, P2a and P3 respectively. Baseline was designated from −200 to 0 ms relative to stimulus onset. ERP analyses were undertaken via 3 (CONDITION; Predictable-A, Predictable-B, Unpredictable) × 4 (STIMULUS; S1, S2, S3, S4) repeated-measures ANOVAs in SPSS 20 (IBM) for each time window of interest. P values were calculated by using the Greenhouse-Geisser correction when appropriate. Post-hoc comparisons were conducted using Bonferroni correction. The level of significance was established in every case in 0.05.

#### Analyses of the oscillatory activity

Spectral changes in oscillatory activity were analysed using a wavelet transform. Trial-by trial time-frequency (TF) analyses was computed for every subject, condition, and sensor separately using a Gaussian-windowed sinusoidal moving Morlet wavelet with linearly increased cycles, from 2 cycles for the lowest frequency (2 Hz) to 15 cycles for the highest frequency (30 Hz) analysed (step size, 0.5 Hz). Changes in event-related spectral power at different time points, relative to power in baseline (from −2000 to −100 ms relative to S1 onset) were computed by the Event-Related Spectral Perturbation (ERSP) index^61^. Significant changes in ERSP are reflected by mean TF power values that exceed the significant cut-off threshold extracted from the baseline period. To determine the threshold significance of ERSP, bootstrap distributions (p < 0.01), extracted randomly from baseline data (−2000 to −500 ms) and applied 200 times, were used^61^. Once the spectral power changes were obtained, differences between conditions in the resulting changes in power (dB) were addressed using permutation analyses (2000 permutations, alpha level 0.05) corrected for multiple comparisons with false discovery rate (fdr). From this point, analyses were conducted following a three-step procedure. Firstly, power values across electrodes, time intervals, and frequency bands where permutations showed significant differences between conditions were identified. In the theta range, this allowed us to pick out differences 280–520 ms post-S2 and post-S3 onset at central sites (4–7 Hz), and 120–360 ms post-S2 onset at parieto-occipital sites (4–6 Hz). In the alpha range, permutations revealed differences between conditions in the 200 ms period preceding S4 onset at central sites between 10–12 Hz. Secondly, sites that showed similar differences between conditions were grouped into ROIs to minimize the influence of individual differences in topographies as well as the effects of performing multiple comparisons. Thus, a central (FC1, FC2, C1, Cz, C2, CP1, CPz and CP2) and a parieto-occipital ROI (P7, PO3, PO7, P8, PO4, PO8, O1, O2) were created to study power modulations in the theta range, and a central (FC1, FC2, C1, Cz, C2, CP1, CPz and CP2) ROI was created to do the same in the alpha band. Finally, trial-by-trial TF analyses were recomputed on those ROIs following the same procedure explained above. Power values across time intervals and frequency bands where permutations showed significant differences between conditions in those ROIs were submitted to parametric analyses by means of separate repeated measures ANOVAs. Since central and parieto-occipital theta power enhancements were observed in response to every stimulus in the sequence, STIMULUS was included as a factor in the statistics with the aim of checking if the significant differences revealed by permutations in response to S2, S3 and S4 were due to those activities being differently modulated throughout the sequence in each condition. Thus, analysis of central theta (4–7 Hz, 280–520 ms post-stimulus) and parieto-occipital theta (4–6 Hz, 120–360 ms post-stimulus) were undertaken via 3 (CONDITION; Predictable-A, Predictable-B, Unpredictable) × 4 (STIMULUS; S1, S2, S3, S4) repeated-measures ANOVA on the central and parieto-occipital ROIs, respectively. Differences between conditions in pre-stimulus central alpha modulations were assessed separately via repeated-measures ANOVA with CONDITION (Predictable-A, Predictable-B, Unpredictable) as within-subject factor. The level of significance was established in every case in 0.05, p values were calculated by using the Greenhouse-Geisser correction when appropriate, and post-hoc comparisons were conducted using Bonferroni test.

### Data availability

The datasets analysed during the current study are available from the corresponding author on request.

## Electronic supplementary material

Supplementary material
