# Supplementary material for: Predictions through evidence accumulation over time
Source: Sci Rep. 2018 Jan 11;8:494. doi: 10.1038/s41598-017-18802-z (PMC5765034; doi:10.1038/s41598-017-18802-z)
Supplement: Supplementary file 1 — Supplementary material [file 41598_2017_18802_MOESM1_ESM.doc]

EquationSupplementary material

Equation **Predictions through evidence accumulation over time**

Álvaro Darriba^1,2^* and Florian Waszak^1,2^

^1^Université Paris Descartes, Sorbonne Paris Cité, 75006 Paris, France

^2^Centre National de la Recherche Scientifique, Laboratoire Psychologie de la Perception, Unité Mixte de Recherche 8242, 75006 Paris, France

Corresponding Author: * alvarodarriba@gmail.com

**Information estimates**

Within the framework of Shannon’s information theory ^1^, we computed surprise and predictive information on the forthcoming stimulus at each time point in the predictable and unpredictable series to quantify PE and prediction build-up through evidence accumulation, respectively. The surprise inherent in an event is based on the probability or, conversely, the uncertainty of that event ^2^, and can be taken as a measure of the PE size ^3^. Predictive information, on the other hand, is a measure of surprise or uncertainty reduction due to the knowledge of the preceding events. It quantifies the amount of information available at a given time for predicting which the forthcoming stimulus will be ^4^. In order to quantify those measures, we used the notion of an ‘ideal’ observer and a simple Bayesian update scheme to estimate the probability of the event i (Eq.1) and of the joint probability of two (i and j), three (i, j, k), and four (i, j, k, l) consecutive events (Eq. 2-4, respectively) ^4,5^. We assumed that, at the beginning of each trial, the observer started with no knowledge on the upcoming sequence. For each new shape e_t_, presented at time step t, those probabilities are defined in the following way:

$prob\left(e_{t}=i\right)=\frac{n_{i}^{t}}{\sum_{i} n_{i}^{t}}$ (1)

$prob\left(e_{t}=i,e_{t-1}=j\right)=\frac{n_{i,j}^{t}}{\sum_{i,j} n_{i,j}^{t}}$ (2)

$prob\left(e_{t}=i,e_{t-1}=j,e_{t-2}=k\right)=\frac{n_{i,j,k}^{t}}{\sum_{i,j,k} n_{i,j,k}^{t}}$ (3)

$prob\left(e_{t}=i,e_{t-1}=j,e_{t-2}=k,e_{t-3}=l\right)=\frac{n_{i,j,k,l}^{t}}{\sum_{i,j,k,l} n_{i,j,k,l}^{t}}$ (4)

where $n_{i,j,k,l}^{t}$ is the number of quartets i, j, k, l at time step t; $n_{i,j,k}^{t}$ is the number of triplets i, j, k at time step t; and $n_{i,j}^{t}$ is the number of duplets i, j at time step t.

Following previous studies ^2,5,6^, we calculated surprise (*S_t_*) driven by each new stimulus *e_t_* displayed at sequence positions S1, S2, S3, and S4 (Eqs. 5-8), for each condition. Except for S1, surprise was calculated from conditional probabilities. Calculations were made as follows (cf. Shannon, 1948):

$S_{t}\left(e_{t}=i\right)=-\log_{2}\left(prob_{t}\left(e_{t}=i\right)\right)$ (5)

$S_{t}\left(e_{t}=i\right)=-\log_{2}\left(prob_{t}\left(e_{t}=i\left|e_{t-1}=j\right.\right)\right)$ (6)

$S_{t}\left(e_{t}=i\right)=-\log_{2}\left(prob_{t}\left(e_{t}=i\left|e_{t-1}=j,e_{t-2}=k\right.\right)\right)$ (7)

$S_{t}\left(e_{t}=i\right)=-\log_{2}\left(prob_{t}\left(e_{t}=i\left|e_{t-1}=j,e_{t-2}=k,e_{t-3}=l\right.\right)\right)$ (8)

We followed Domenech and colleagues ^4^ for computing the predictive information conveyed by the preceding event (abbreviated as p_1,t_ in Eq. 9), by the two preceding events (abbreviated as p_2,t_ in Eq. 10), and by the three precedent events (p_3,t_ in Eq. 11), depending on the position of the stimulus in the sequence. For each new stimulus e_t_, displayed at the time step t, current estimates of the predictive information were defined in the following way:

$p_{1,t}\left(e_{t}=i,e_{t-1}=j\right)=\log_{2}\left(\frac{prob_{t}\left(e_{t}=i\left|e_{t-1}=j\right.\right)}{prob_{t}\left(e_{t}=i\right)}\right)$ (9)

$p_{2,t}\left(e_{t}=i,e_{t-1}=j,e_{t-2}=k\right)=\log_{2}\left(\frac{prob_{t}\left(e_{t}=i\left|e_{t-1}=j,e_{t-2}=k\right.\right)}{prob_{t}\left(e_{t}=i\right)}\right)$ (10)

$p_{3,t}\left(e_{t}=i,e_{t-1}=j,e_{t-2}=k,e_{t-3}=l\right)=\log_{2}\left(\frac{prob_{t}\left(e_{t}=i\left|e_{t-1}=j,e_{t-2}=k,e_{t-3}=l\right.\right)}{prob_{t}\left(e_{t}=i\right)}\right)$ (11)

**References**

1. Shannon, C. E. A Mathematical Theory of Communication. *Bell System Technical Journal* **27,** 379–423 (1948).

2. Strange, B. A., Duggins, A., Penny, W., Dolan, R. J. & Friston, K. J. Information theory, novelty and hippocampal responses: unpredicted or unpredictable? *Neural Networks* **18,** 225–230 (2005).

3. Ouden, den, H. E. M., Friston, K. J., Daw, N. D., McIntosh, A. R. & Stephan, K. E. A Dual Role for Prediction Error in Associative Learning. *Cerebral Cortex* **19,** 1175–1185 (2009).

4. Domenech, P. & Dreher, J.-C. Decision Threshold Modulation in the Human Brain. *Journal of Neuroscience* **30,** 14305–14317 (2010).

5. Harrison, L. M., Duggins, A. & Friston, K. J. Encoding uncertainty in the hippocampus. *Neural Networks* **19,** 535–546 (2006).

6. Mars, R. B. *et al.* Trial-by-Trial Fluctuations in the Event-Related Electroencephalogram Reflect Dynamic Changes in the Degree of Surprise. *Journal of Neuroscience* **28,** 12539–12545 (2008).
